# Reference Genes Selection and Validation for *Cinnamomum burmanni* by Real-Time Quantitative Polymerase Chain Reaction

**DOI:** 10.3390/ijms25063500

**Published:** 2024-03-20

**Authors:** Lingling Shi, Yanling Cai, Jun Yao, Qian Zhang, Boxiang He, Shanzhi Lin

**Affiliations:** 1 College of Biological Sciences and Biotechnology, National Engineering Laboratory for Tree Breeding, Key Laboratory of Genetics and Breeding in Forest Trees and Ornamental Plants, Ministry of Education, Tree and Ornamental Plant Breeding and Biotechnology Laboratory of National Forestry and Grassland Administration, Beijing Forestry University, Beijing 100083, China; m15833409013@163.com (L.S.); caiyl@sinogaf.cn (Y.C.); 2 Guangdong Provincial Key Laboratory of Silviculture, Protection and Utilization, Guangdong Academy of Forestry, Guangzhou 510520, China; yaojun@sinogaf.cn (J.Y.); zhangq7610@sinogaf.cn (Q.Z.)

**Keywords:** q-PCR, normalization, *Cinnamomum burmannii*, reference gene

## Abstract

In recent years, the field of biology has witnessed a surge of interest in genomics research due to the advancements in biotechnology. Gene expression pattern analysis plays a crucial role in this research, as it enables us to understand the regulatory mechanism of gene expression and the associated biological processes. Real-time quantitative polymerase chain reaction (q-PCR) is an efficient method to analyze the gene expression patterns, for which accuracy relies on the standardized analysis of reference genes. However, numerous studies have shown that no reference gene is universal in all conditions, so screening a suitable reference gene under certain conditions is of great importance. *Cinnamomum burmannii* (*C. burmannii*) is rich in volatile components and has high medicinal and economic value. However, knowledge of the screening of reference genes for the gene expression analysis of *C. burmannii* is insufficient. Aiming at this problem, we evaluated and screened the reference genes in *C. burmannii* under different experimental conditions, including different abiotic stresses (Cold-treated, PEG-treated and Nacl-treated), different tissues, leaves at different developmental stages and different chemical types. In this study, different algorithms (∆Ct, geNorm, NormFinder and BestKeeper) were used to evaluate the stability of the candidate reference genes, and RefFinder further merged the output data to screen out the optimum reference gene under various experimental conditions in *C. burmannii*. The results showed that the optimal reference gene number for gene standardization was 2 under different experimental conditions. *RPL27*|*RPS15* was the most suitable combination under the Nacl-treated and PEG-treated samples. *RPL27*|*APT* was the optimum combination under the Cold-treated samples. The optimal combinations of other samples were *EF1α*|*ACT7* for different tissues, *eIF*-*5A*|*Gllα* for different borneol clones in *C. burmannii*, *RPS15*|*ACT7* for leaves at different developmental stages and *RPS15*|*TATA* for all samples. Additionally, two terpenoid synthesis-related genes (*CbWRKY4* and *CbDXS2*) were standardized to verify the feasibility of the selected reference genes under different experimental conditions. This study will be helpful for the subsequent molecular genetic mechanism study of *C. burmannii*.

## 1. Introduction

Real-time quantitative polymerase chain reaction (q-PCR) is a widely utilized method in molecular biology for investigating gene expression differences across various cell types, tissues, organs or developmental stages [1,2,3]. By comparing gene expression in different samples, researchers can identify genes that are activated or inhibited under specific physiological or pathological conditions, which is of great significance for studying the mechanisms of human diseases, finding treatments and improving crop traits. Compared with traditional PCR, a q-PCR has the advantages of rapidity, sensitivity, specificity and quantification [4]. However, the reliability of a q-PCR is affected by many factors, such as RNA quality, PCR amplification efficiency and differences between samples [5]. Currently, the most common method for correcting and standardizing the q-PCR data is to select appropriate reference genes [6]. Reference genes are expressed in various cells of organisms, and their products are proteins necessary to maintain the basic life activities of cells. Ideally, the expression level of a selected reference gene should be relatively constant in various tissues, cells and experimental conditions. However, multiple forms of evidence suggested that it was difficult to have a single reference gene that was universal for all conditions. For instance, glycerol-aldehyde-3-phosphate dehydrogenase (*GAPDH*), a commonly used reference gene, was selected as having the best stability in *Carex rigescens* under salt-treated leaves [7] but not suitable for *Salsola ferganica* under six abiotic stresses [8] and *Betula platyphylla* under salt and osmotic stress conditions [9]. In addition, actin (*ACT*) was selected as the optimum reference gene for leaves in *Solanum lycopersicum* exposed to UV-B María [10], but its stability was poor in most experiments with pecan [11]. Such phenomena have urged more and more studies to focus on the screening of reference genes in biological samples under certain circumstances. At present, attempts have been made to screen reliable reference genes for a q-PCR analysis in many plants, such as sweetpotato [12], Siberian Apricot [13], Sorghum [14], pecan [11], *Metasequoia* [15], *Rubus* [16], *Schima superba* [17,18], etc. 

*Cinnamomum burmannii* (*C. burmnnii*), a *Cinnamomum* species in Lauraceae, is an important aromatic medicinal and green tree species, mainly distributed in the Guangdong, Guangxi and Fujian provinces in China. *C. burmannii* leaves contain a variety of volatile compounds and extensive research showed that *C. burmannii* had potential health benefits, such as antibacterial, antioxidant, antidiabetic and antitumor [19,20,21,22]. In particular, the borneol-type essential oil is an important raw material for cosmetics and medicine due to the better permeability and antibacterial properties of borneol [23]. In the past few decades, the studies of *C. burmannii* mainly focused on the extraction, composition analysis of the compounds and biological activity [21,24,25,26], but there is only limited research on gene regulation [27,28,29]. To some extent, this hindered the genetic improvement of the crop, and understanding the biological function of this crop is very important for further molecular breeding. An accurate gene expression analysis will provide a powerful and valuable approach to understand the molecular biological mechanisms of growth and development, as well as signal transduction and metabolism [30,31]. However, to our best knowledge, there is no report on the systematic reference gene screening of *C. burmannii*. Hence, it is very necessary to study the reference gene selection of *C. burmannii* in order to improve the reliability of the gene expression analysis. 

In this study, the stability of 13 candidate reference genes was evaluated under a series of experimental conditions. In order to verify the reliability and accuracy of the reference genes, the expression trends of two terpenoid synthesis-related genes *CbWRKY4* and *CbDXS2* were detected under different experimental conditions. Terpenoids are a kind of natural compound that exist widely in nature and have great value to plants, animals and humans. As the first enzyme of the MEP pathway, 1-deoxyxylose-5-phosphate synthetase (DXS) is a rate-limiting enzyme of this pathway, which plays a key role in regulating the synthesis of terpenoids [32]. WRKY is a class of DNA-specific binding transcription factors that regulate metabolic processes by binding promoter elements of key enzyme genes in the plant secondary metabolic biosynthesis pathway [33]. The reference genes identified through this study will facilitate the future gene function analysis in *C. burmannii.*

## 2. Results

### 2.1. Primer Specificity and Amplification Efficiency of Candidate Reference Genes

The agarose gel electrophoresis results showed that the PCR amplification product of the reference genes was consistent with the expected size and had a single band (Figure S1). A q-PCR analysis showed that each pair of primers had a single peak (Figure S2). The amplification efficiency (E) and the regression coefficient values R^2^ of each pair of primers are shown in Table 1. All the results suggested that the candidate gene primers used in this study can be used for further q-PCR analysis. 

### 2.2. Expression Analysis of Candidate Reference Genes of C. burmannii

**Figure 1:**
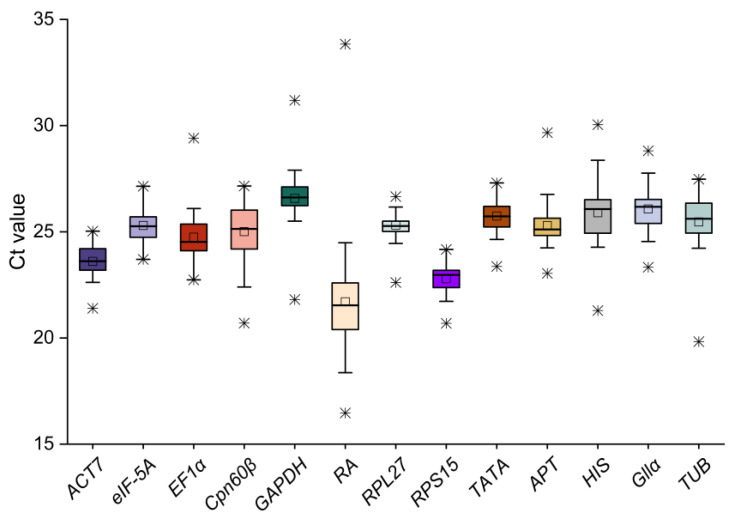
The Ct values of the candidate reference genes in all the materials. The boxes indicate the 25th and 75th percentiles in all the samples. The square represents the median. The * represents the maximum and minimum values.

**Figure 2:**
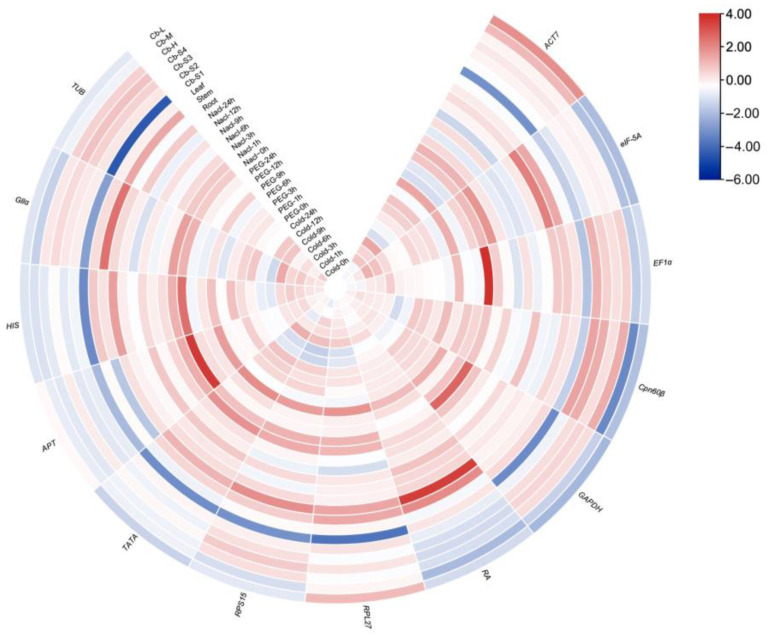
Heat map of expression levels of reference genes in all samples.

The transcriptional levels of the candidate reference genes in different materials were determined by the Ct values, and the gene expression varied from sample to sample (Figure 1). Among these, the expression level of *RA* was the highest with the mean Ct (21.70) across all materials, while the expression abundance of *GAPDH* was the lowest with the mean Ct (26.57). The results suggested that for the gene expression level exists obvious divergence in all the samples. Meanwhile, the transcription level of the reference genes also showed different expression variation, and *ACT7*, *RPL27*, *RPS15*, *TATA* and *eIF*-*5A* had a relative narrower Ct range, indicating that these genes might be expressed more stably. Furthermore, the Log2 Fold method was used to calculate the expression levels of the candidate genes in all the materials to analyze their expression stability, and the heat map clearly shows the expression level of each gene in each sample (Figure 2).

### 2.3. Gene Expression Stability Analysis

The stability of the reference gene was evaluated by ∆Ct, and the gene associated with the lowest mean standard deviation (mSD) was thought to be the optimum. The results of the ∆Ct analysis showed that *RPS15* was the most stable gene in Nacl-treated, PEG-treated, leaves at different developmental stages, different borneol clones and total samples (Table 2). *RPL27* was the most stable gene in the Cold-treated samples and *EF1α* had the best stability among the different tissues.

**Figure 3:**
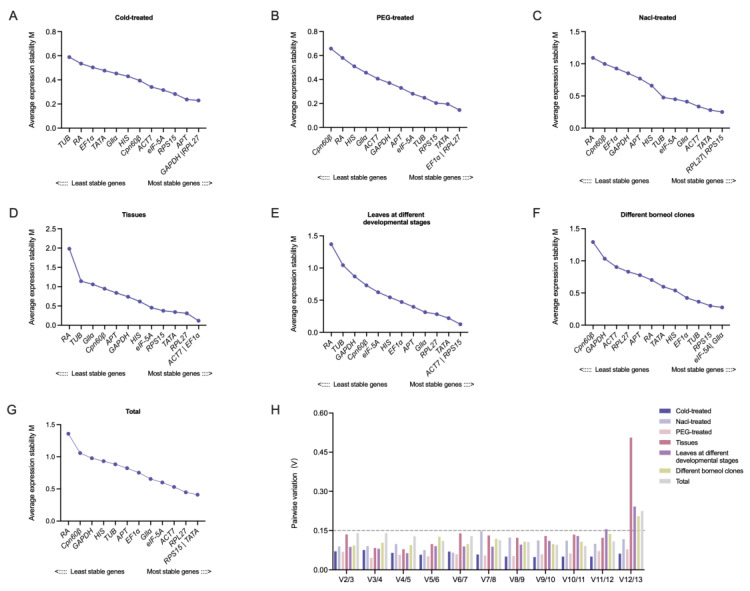
Gene stability values of reference genes and determination of the optimum number of reference genes for q-PCR based on geNorm in different experiment conditions. (**A**): Cold-treated samples; (**B**): PEG-treated samples; (**C**): Nacl-treated samples; (**D**): different tissues; (**E**): leaves at different developmental stages; (**F**): different borneol clones; (**G**): total samples; and (**H**): the pairwise variation (Vn/n + 1) was analyzed between the normalization factors to determine the optimal number of reference genes for q-PCR normalization by geNorm.

Meanwhile, geNorm analyzed the expression stability of the 13 candidate genes according to the M value (threshold value was 1.5) (Table S1 and Figure 3). The candidate genes with M < 1.5 could be used for the standardized analysis, and the smaller the M value, the better the gene stability. In this study, the lowest M of *RPL27*|*RPS15* in the Nacl-treated samples indicated the highest stability, while the highest M of *RA* indicated the lowest stability. In the PEG-treated samples, *EF1α*|*RPL27* showed the most stable expression, and *Cpn60β* was the most unstable. *GAPDH*|*RPL27* was the most suitable combination in the Cold-treated samples, while *TUB* was the least suitable. In the plant tissues, *ACT7*|*EF1α* was the optimum combination and *RA* was the poor one. The stability of *eIF-5A*|*Gllα* was higher than that of the other genes in different borneol clones. *ACT7*|*RPS15* was the best rank in the leaves at different developmental stages, while *RA* was the worst. After a comprehensive evaluation of all the samples, the stability of *RPS15*|*TATA* was the best, while that of *RA* was the worst. In addition to determining the expression stability of the candidate reference genes, geNorm could also determine the optimum number of reference genes by analyzing the pairwise variation (Vn/Vn + 1). In this study, the V2/3 were all less than 0.15, indicating that the standardized analysis of the q-PCR in *C. burmannii* could be met by using two reference genes (Figure 3H).

Furthermore, NormFinder further determined the stability of the candidate genes via SV and a lower SV indicated more stability (Table 3). In the Nacl-treated samples, *RPL27* (0.328) was expressed most stably, with *RA* (1.499) the most unstable. In the PEG-treated samples, *RPS15* (0.082) was stable, and *Cpn60β* (1.005) was the least stable. In the Cold-treated samples, *RPL27* (0.115) was the optimum, and *TUB* (0.803) was expressed most unstably. *EF1α* (0.058) was expressed most stably in the different tissues, while *RA* (6.568) expression was the most unstable. In different borneol clones, the stability of *eIF*-*5A* (0.128) was most stable, and *Cpn60β* (2.66) was the most unstable. *RPS15* (0.063) was expressed most stably in the leaves at different developmental stages, and *RA* (3.132) was the most unstable. The NormFinder analysis of all the samples showed that *RPS15* (0.21) was expressed most stably and *RA* (2.918) was the least stable.

Moreover, BestKeeper calculated the standard coefficient of variation (SD) and coefficient of variation correlation (CV) of the Ct values of all the candidate genes, and the relatively low SD values (less than 1) were generally considered to be in the acceptable range (Table 4). In the Nacl-treated samples, *TATA* (0.38) was the most stable, while *EF1α* (1.28) was the least stable. In the PEG-treated samples, *RPL27* (0.35) was stable, while *RA* (0.8) was the least stable. In the Cold-treated samples, *APT* (0.27) was expressed most stably, but the expression of *HIS* (0.74) was the most unstable. *ACT7* (0.19) was expressed stably in the different tissues, and the expression of *RA* (4.74) was the most unstable. Among the different borneol clones, *HIS* (0.1) ranked the best, while *Cpn60β* (2.37) ranked the worst. In the leaves at different developmental stages, *eIF*-*5A* (0.46) was the most stable, while *TUB* (2.38) was the most unstable. The BestKeeper analysis of all the samples showed that *RPL27* (0.47) was the most stable, while *RA* (1.87) was the least stable.

**Figure 4:**
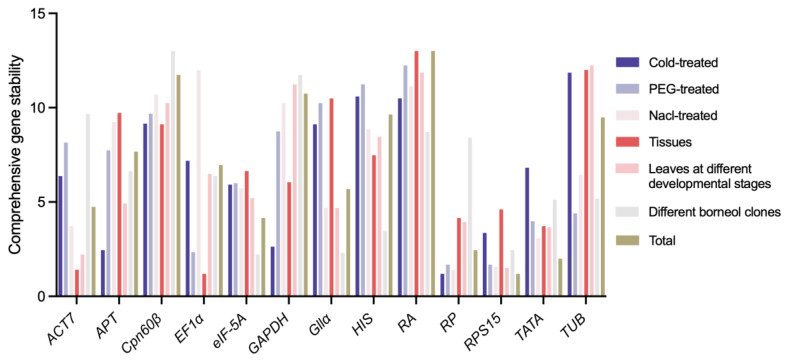
Comprehensive stability analysis of reference genes based on RefFinder in different experiment conditions.

Ultimately, RefFinder further merged the output data to screen out the optimum reference gene in the different experimental materials (Figure 4). The expression stability of *RPL27*|*RPS15* was higher than that of the other genes in the Nacl-treated and PEG-treated samples. *RPL27*|*APT* ranked best in the Cold-treated samples and *EF1α*|*ACT7* was the most suitable combination in the different tissues. *eIF*-*5A*|*Gllα* was suitable for different borneol clones and *RPS15*|*ACT7* was the optimum in leaves at different developmental stages. When analyzing all the samples, *RPS15*|*TATA* was the best combination in all the samples. The Ct values of all the candidate reference genes in various materials for reference gene screening can be found in Table S2 of the Supplementary Material.

### 2.4. Reference Gene Validation

**Figure 5:**
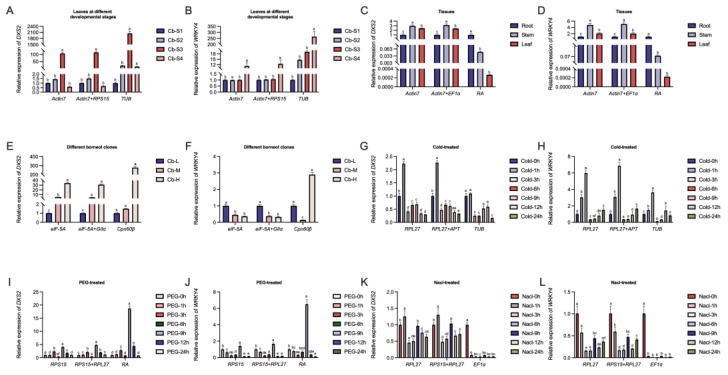
Normalization of relative expression levels of *CbWRKY4* and *CbDXS2* using the identified reference gene. (**A**): *CbDXS2* for leaves at different developmental stages; (**B**): *CbWRKY4* for leaves at different developmental stages; (**C**): *CbDXS2* for tissues; (**D**): *CbWRKY4* for tissues; (**E**): *CbDXS2* for different borneol clones; (**F**): *CbWRKY4* for different borneol clones; (**G**): *CbDXS2* for Cold-treated samples; (**H**): *CbWRKY4* for Cold-treated samples; (**I**): *CbDXS2* for PEG-treated samples; (**J**): *CbWRKY4* for PEG-treated samples; (**K**): *CbDXS2* for Nacl-treated samples; and (**L**): *CbWRKY4* for Nacl-treated samples. a, b, c, d, e and f indicate significant differences at *p* < 0.05.

To verify the accuracy and suitability of the selected reference genes, the expression levels of two terpenoid synthesis-related genes (*CbWRKY4* and *CbDXS2*) were evaluated using two stable reference genes and the unstable reference gene under different experimental conditions. DXS, a key rate-limiting enzyme, is pivotal in the MEP pathway for terpenoid synthesis and exerts influence on the downstream metabolite content [34,35]. WRKY transcription factors play a crucial role in terpenoid synthesis by specifically binding to the promoter elements of key genes involved in the terpenoid synthesis pathway [36,37]. Our results show that the expression patterns of *CbDXS2* and *CbWRKY4* differ significantly using different reference genes for q-PCR normalization in all the experimental treatments (Figure 5). The expression patterns of *CbDXS2* and *CbWRKY4* were similar using the optimal and the best combination reference genes. However, after the normalization of the unstable reference genes, the expression patterns of *CbDXS2* and *CbWRKY4* were significantly different from those of the optimal reference gene combination. For instance, the expression levels of *CbDXS2* and *CbWRKY4* in roots were the lowest when normalized by *ACT7* and *EF1α*, while the expression levels of *CbDXS2* and *CbWRKY4* in roots were the highest when normalized by *RA* in different tissues (Figure 5C,D). *CbDXS2* had the highest expression at 1 h using *RPL27* and *RPS15* for q-PCR normalization, while *CbDXS2* was hardly expressed at 1 h using *EF1α* under the Nacl-treated samples (Figure 5K). All the results showed that the selection of appropriate reference genes was crucial for the accurate normalization of gene expression.

## 3. Discussion

Nowadays, a q-PCR is regarded as an efficient tool to understand the molecular biology research [38,39], for which accuracy relies on the normalization of reference genes [40]. However, numerous studies have shown that the gene expression level of a reference gene differs in different experimental conditions [41,42,43], which was also confirmed in our study where the stability of 13 reference genes was different under certain conditions. Therefore, screening appropriate reference genes under specific conditions is of great significance for subsequent gene expression analysis. The identification of appropriate reference genes in *C. burmannii* will promote the study of the gene regulation of this species.

In this study, ∆Ct [44], BestKeeper [6], geNorm [45] and NormFinder [46] were used to evaluate the candidate reference genes in *C. burmannii* under different experimental conditions. The results demonstrated that there were some differences in the stability of the reference genes among the different software. The reason for this may be due to the differences in algorithms between the software [47] and the similar phenomena were often seen in other research, such as in Carex rigescens [7], Luffa [48] and *Rubia yunnanensis Diels* [49]. In this case, a further comprehensive analysis of the results based on the geometric means of the results to reduce the bias caused by differences in the software algorithms better reflects the expression stability of the reference genes under certain conditions (Figure 4). Considering the reliability and accuracy of q-PCR normalization, a growing number of studies showed that a single reference gene sometimes cannot guarantee the accuracy of experimental results; two or more reference genes were needed for a q-PCR standardized analysis [50,51]. In this study, the comprehensive verification analysis showed that two reference genes could meet the requirements of a q-PCR normalization analysis (Figure 3H). 

According to the results of the stability evaluation in this study, no reference gene was suitable for all experimental conditions. Under most experimental conditions, ribosomal proteins (RPs) showed good expression stability, for example, *RPS15* was the most stably expressed in the total sample, PEG-treated sample and leaves at different developmental stages, and *RPL27* showed relatively high stability in the Cold-treated and Nacl-treated samples (Figure 4). As genes encoding ribosomal protein, RPs have an important role in cellular protein biosynthesis, and previous studies also identified RPs as the reference genes, such as *RPL19* for potato tissues [52], *RPL5* for MeJA, cold and hot stress in *Rubia yunnanensis* Diels [49] and *RPS15* for developmental stages, *RPL32* for tissues and temperature stress and *RPS3* for insecticide stress and starvation stress in Lymantria dispar [53]. Actin is highly conserved and expressed in almost all eukaryotic cells [54] and is usually used as a reference gene for q-PCR normalization. However, in this study, *ACT7* was the proper gene only under specific conditions; just like in Scutellaria baicalensis Georgi, *ACT7* showed high stability under hormonal conditions but was not the best choice in other conditions [42], and in Haloxylon ammodendron, *ACT7* was stable under salt treatment but poor under other conditions [55]. In addition, we compared the expression levels of *AtACT2* (*Arabidopsis*) with those of *ACT7* and the stable reference genes *RPL27* and *RPS15* (*C. burmnnii*) under the Nacl-treated samples (Figure S3), showing that the stability of *AtACT2* was relatively lower. Based on the previous research, *TATA-box*, as the first promoter found in eukaryotes, was more suitable for q−PCR analysis in a variety of species, such as in *Monomorium pharaonic* [1], *Gleditsia microphylla* [56] and *Dendrobium huoshanense* [57], but in our study it was not the optimum reference gene for some experimental conditions. In addition, *eIF*-*5A* was just the best reference gene in different borneol clones and *EF1α* was the optimum gene for studying different tissues. Moreover, the common reference gene *GAPDH* was highly stable in many species [58,59,60], but the stability was not as expected in this study, indicating that the reference gene needed to be re-screeded in different species. All the results suggested the importance of a suitable reference gene for the gene function research, and it was of great significance to evaluate and screen reference genes under certain experimental conditions.

A validation experiment is the prerequisite to evaluate the accuracy and stability of reference genes. Therefore, we examined the expression trends of two terpenoid synthesis-related genes *CbWRKY4* and *CbDXS2* under different experimental conditions to determine the accuracy of the selected reference genes. The results in Figure 5 showed that the expression patterns of target genes were significantly different after the normalization using the stable reference gene and the unstable reference gene under different experimental conditions. There was little difference in the expression levels when the stable reference genes were normalized alone or in combination, while the least stable reference genes were normalized with greater difference in the expression levels. This phenomenon further revealed that the reliable gene expression analysis depended on the stable reference gene and the necessity of screening reference genes for the accuracy of q-PCR results. This process of reference gene screening under various experiment conditions can provide guidance for researchers to study the genetic breeding of *C. burmannii*.

## 4. Materials and Methods

### 4.1. Plant Materials 

*C. burmannii* was obtained from a nursery managed by the Guangdong Academy of Forestry. Seedlings of 1–2 years were selected for cultivation in an artificial climate chamber (light/dark = 16 h/8 h and relative humidity = 65–75%). To induce different abiotic stress conditions, the seedlings were exposed to various treatments. Seedlings treated with cold were grown at 16 °C, and those treated with 200 mM NaCl and 20% PEG 6000 were grown at 25 °C. Leaves from all abiotically stressed seedlings were collected at 0, 1, 3, 6, 9, 12 and 24 h after treatment. Samples of plant tissues were collected from distinct parts of the plant, encompassing mature leaves, stems and roots. Mature leaves from different borneol clones of *C. burmannii* (Cb-H, 51.96%; Cb-M, 27.65%; and Cb-L, 0.00%) were collected. Leaves at different developmental stages (Cb-S1, Cb-S2, Cb-S3 and Cb-S4) were collected from the same material, in accordance with our previous research [27]. All the samples were frozen immediately in liquid nitrogen and stored at −80 °C and all the treatments were conducted in triplicate. 

### 4.2. RNA Extraction and cDNA Synthesis

The total RNA was extracted using an RNAprep Pure Plant kit (Polysaccharides and Polyphenolics rich) (Tiangen, Beijing, China). The RNA integrity and purity was determined by 1% agarose gel electrophoresis and OD_260/280_. cDNA was synthesized using a PrimeScript™ RT reagent Kit with gDNA Eraser (Perfect Real Time) (Takara, Beijing, China) and stored at −20 °C for the subsequent q-PCR analysis.

### 4.3. Candidate Reference Genes Selection and Primer Design

The candidate reference genes were selected based on our previous transcriptome and other common reference genes information. Primer Premier 5.0 was used to design the primers for q-PCR (Table 1), and the primer design criteria were G + C (40–60%), PCR product (80–300 bp), TM (58–62 °C), and primer length (17–25 bp). The specificity of each primer was verified by 1% agarose gel electrophoresis and melting curve. The amplification efficiency (E) of the candidate genes was calculated using a standard curve (a 5-fold dilution series cDNA was used as the template) by q-PCR. E (%) = (10^−1/slope^ –1) ×100% [61].

### 4.4. q-PCR Amplification

A q-PCR was performed on CFX Connect^TM^ real-time systems (Bio-Rad, Singapore) with Biomike fluorescent quantitative SYBR reagent under the following reaction system: Biomarker 2× SYBR Green Fast qPCR MIX (10 μL), Forward Primer (0.4 μL), Reverse Primer (0.4 μL), cDNA (1 μL), and Nuclease-free H_2_O (8.2 μL). The reaction conditions were as follows: 95 °C for 3 min; 40 cycles: 95 °C for 5 s and 60 °C for 30 s; melting curve: instrument default. Three techniques were repeated for each sample.

### 4.5. Data Analysis and Validation of Selected Reference Genes 

The stability of the candidate reference genes was assessed using different algorithms: ∆Ct [44], BestKeeper [6], geNorm [45], NormFinder [46] and RefFinder [62]. The ∆Ct method calculates the average standard deviation (SD) of all potential reference gene pairings, with the gene displaying the lowest SD considered the most stable. The algorithms of geNorm and NormFinder rely on the transformation of Ct values into 2^−∆Ct^ values. geNorm is utilized for evaluating the stability of reference genes through the calculation of the M value, where a lower M value suggests better stability. Furthermore, geNorm is capable of determining the optimal number of normalization genes. NormFinder evaluates the expression stability of candidate genes by calculating the stability value (SV), where the lower SV of the reference gene indicates greater stability. In contrast to geNorm and NormFinder, the analysis conducted by BestKeeper utilizes the Ct values in order to calculate the standard deviation (SD) and coefficient of variance (CV). A smaller SD value indicates a higher level of stability in the expression of reference genes. The RefFinder is utilized to conduct a comparative analysis of the aforementioned data. The final overall ranking is determined by RefFinder through calculating the geometric mean, which helps identify the optimal reference gene. Finally, two terpenoid synthesis-related genes (*CbWRYK4* and *CbDXS2*) were analyzed to verify the reliability and suitability of the selected reference genes under the above different conditions. 

## 5. Conclusions

In this study, the expression stability of 13 candidate genes under different experimental conditions was evaluated for a standardized q-PCR analysis of *C. burmanni*. ∆Ct, geNorm, NormFinder and BestKeeper were used to evaluate the gene stability, and the results were further ranked based on the geometric mean to screen out the optimum reference genes in the diverse experimental conditions. The expression stability of *RPL27*|*RPS15* was higher than that of the other genes in the Nacl-treated and PEG-treated samples. *RPL27*|*APT* ranked best in the Cold-treated samples and *EF1α*|*ACT7* was the most suitable combination in different tissues. *eIF*-*5A*|*Gllα* was suitable for different borneol clones and *RPS15*|*ACT7* was the optimum in leaves at different developmental stages. In all the samples, *RPS15*|*TATA* was the best combination. All the results suggested the importance of selecting appropriate reference genes under specific experimental conditions for q-PCR analysis. This study will contribute to the subsequent research on the genetic molecular mechanism and genetic breeding of *C. burmannii.*

## Figures and Tables

**Table 1:** Primer sequences and PCR amplification characteristics of 13 reference genes.

Gene-ID	Gene Abbreviation	Tentative Annotation	Primer Sequence of Forward	Primer Sequence of Reward	Amplicon Length (bp)	Tm (°C)	E	R^2^
Cbur01G028330	*ACT7*	actin7	CAACCCAAAAGCCAACAGG	TCACCCGAGTCCAGAACAATAC	141	58.7/59.1	98.76%	0.9968
Cbur02G019900	*Cpn60β*	chaperonin 60 subunit beta 2	CAACAAGGATGGGCTGGCTA	TTGGCCACAGTCACTCCATC	156	60/60	98.05%	0.9979
Cbur01G001170	*EF1α*	elongation factor 1-alpha	GGTACAAGGGCCCAACTCTC	CTGGAGAGCTTCATGGTGCA	236	60/60	89.99%	0.9983
Cbur05G032970	*eIF*-*5A*	eukaryotic translation initiation factor 5A	CCAAGTGTCACTTTGTGGCG	AGTGGGGAGCCTCAGATCAT	191	60/60	86.05%	0.9993
Cbur10G024220	*GAPDH*	glyceraldehyde-3-phosphate dehydrogenase	AAGGGTGGTGCCAAGAAAGT	GTTGCAGTGATGGAGTGGACAG	215	58.6/60.2	92.81%	0.9917
Cbur06G016220	*GIIα*	glucan 1,3-alpha-glucosidase	CCTTATCGCCTTTTCAACCTT	AGCGTATCAATCCGCCCTC	221	58.3/59.9	90.63%	0.9983
Cbur08G011150	*HIS*	histone superfamily protein H3	GGAGGGAAGGCTCCTAGGAA	CAACTGTTCCAGGGCGGTAT	106	60/60	96.01%	0.9985
Cbur10G000690	*RA*	rubisco activase	ACAGACCGACAAGGACAAATGG	CGGAGACCCGTGCTCAAGTAT	168	61.3/61.6	79.95%	0.9926
Cbur10G003920	*RPL27*	ribosomal protein L27	GCCGTCATCGTACGATCCTT	TGCCGTCTTCTTTGCAGAGT	123	60.0/59.9	98.39%	0.9969
Cbur07G013210	*RPS15*	ribosomal protein S15	GCAGCCGAAGAGGAGAACA	GGCTTCCGCTTCAAACCAC	144	58.4//60.4	92.04%	0.9972
Cbur04G009020	*TATA*	TATA-box-binding protein	CCGTAATGCAGAGTATAACCCC	TTTGACATCACAAGAGCCCAC	146	60.1/59.5	82.13%	0.9989
Cbur08G006150	*TUB*	tubulin β chain	TGGGAATAACTGGGCTAAGGG	AAGCATCATCCGATCAGGGTA	205	60.9/59.5	95.11%	0.9964
Cbur02G028660	*APT*	adenine phosphoribosy ltransferase 1	TGCTTGATCCCGAGGCATTT	ACTTCGAACCAAGGGCCAAA	141	60.1/60	89.03%	0.9993

**Table 2:** Stability evaluation of 13 reference genes analyzed using ∆Ct.

Total		Cold-treated		Nacl-treated		PEG-treated		Tissues		Leaves at Different Developmental Stages		Different Borneol Clones	
Gene	mSD	Gene	mSD	Gene	mSD	Gene	mSD	Gene	mSD	Gene	mSD	Gene	mSD
*ACT7*	1.21	*ACT7*	0.51	*ACT7*	0.87	*ACT7*	0.62	*ACT7*	1.30	*ACT7*	0.94	*ACT7*	1.56
*APT*	1.29	*APT*	0.49	*APT*	1.14	*APT*	0.64	*APT*	1.93	*APT*	1.14	*APT*	1.22
*Cpn60β*	1.61	*Cpn60β*	0.63	*Cpn60β*	1.37	*Cpn60β*	1.09	*Cpn60β*	1.81	*Cpn60β*	1.43	*Cpn60β*	2.72
*EF1α*	1.22	*EF1α*	0.63	*EF1α*	1.41	*EF1α*	0.49	*EF1α*	1.29	*EF1α*	1.10	*EF1α*	1.10
*eIF*-*5A*	1.10	*eIF*-*5A*	0.58	*eIF*-*5A*	0.95	*eIF*-*5A*	0.55	*eIF*-*5A*	1.52	*eIF*-*5A*	1.32	*eIF*-*5A*	0.94
*GAPDH*	1.37	*GAPDH*	0.49	*GAPDH*	1.24	*GAPDH*	0.68	*GAPDH*	1.67	*GAPDH*	1.67	*GAPDH*	1.69
*Gllα*	1.13	*Gllα*	0.62	*Gllα*	0.90	*Gllα*	0.75	*Gllα*	1.91	*Gllα*	0.94	*Gllα*	0.93
*HIS*	1.31	*HIS*	0.66	*HIS*	1.14	*HIS*	0.81	*HIS*	1.73	*HIS*	1.14	*HIS*	1.01
*RA*	3.01	*RA*	0.71	*RA*	1.61	*RA*	0.98	*RA*	6.61	*RA*	3.17	*RA*	1.26
*RPL27*	1.05	*RPL27*	0.43	*RP L27*	0.85	*RP L27*	0.48	*RP L27*	1.32	*RPL27*	0.93	*RPL27*	1.30
*RPS15*	0.98	*RPS15*	0.46	*RPS15*	0.83	*RPS15*	0.47	*RPS15*	1.36	*RPS15*	0.93	*RPS15*	0.90
*TATA*	1.04	*TATA*	0.57	*TATA*	0.92	*TATA*	0.49	*TATA*	1.31	*TATA*	0.95	*TATA*	1.11
*TUB*	1.34	*TUB*	0.89	*TUB*	0.97	*TUB*	0.51	*TUB*	2.03	*TUB*	2.16	*TUB*	1.05

**Table 3:** Stability evaluation of 13 reference genes based on NormFinder.

Total		Cold-treated		Nacl-treated		PEG-treated		Tissues		Leaves at Different Developmental Stages		Different Borneol Clones	
Gene	SV	Gene	SV	Gene	SV	Gene	SV	Gene	SV	Gene	SV	Gene	SV
*ACT7*	0.811	*ACT7*	0.285	*ACT7*	0.438	*ACT7*	0.401	*ACT7*	0.058	*ACT7*	0.063	*ACT7*	1.482
*APT*	0.877	*APT*	0.247	*APT*	0.934	*APT*	0.448	*APT*	1.380	*APT*	0.547	*APT*	1.007
*Cpn60β*	1.314	*Cpn60β*	0.506	*Cpn60β*	1.114	*Cpn60β*	1.005	*Cpn60β*	1.445	*Cpn60β*	1.034	*Cpn60β*	2.660
*EF1α*	0.745	*EF1α*	0.476	*EF1α*	1.311	*EF1α*	0.102	*EF1α*	0.058	*EF1α*	0.439	*EF1α*	0.499
*eIF*-*5A*	0.436	*eIF*-*5A*	0.427	*eIF*-*5A*	0.559	*eIF*-*5A*	0.308	*eIF*-*5A*	0.208	*eIF*-*5A*	0.834	*eIF*-*5A*	0.128
*GAPDH*	0.995	*GAPDH*	0.255	*GAPDH*	1.082	*GAPDH*	0.521	*GAPDH*	1.205	*GAPDH*	1.448	*GAPDH*	1.440
*Gllα*	0.564	*Gllα*	0.473	*Gllα*	0.429	*Gllα*	0.603	*Gllα*	1.390	*Gllα*	0.131	*Gllα*	0.138
*HIS*	0.834	*HIS*	0.545	*HIS*	0.899	*HIS*	0.673	*HIS*	0.948	*HIS*	0.617	*HIS*	0.542
*RA*	2.918	*RA*	0.593	*RA*	1.499	*RA*	0.887	*RA*	6.568	*RA*	3.132	*RA*	0.760
*RPL27*	0.467	*RPL27*	0.115	*RPL27*	0.328	*RPL27*	0.114	*RPL27*	0.126	*RPL27*	0.131	*RPL27*	1.134
*RPS15*	0.210	*RPS15*	0.171	*RPS15*	0.340	*RPS15*	0.082	*RPS15*	0.099	*RPS15*	0.063	*RPS15*	0.153
*TATA*	0.344	*TATA*	0.378	*TATA*	0.570	*TATA*	0.107	*TATA*	0.099	*TATA*	0.119	*TATA*	0.700
*TUB*	0.969	*TUB*	0.803	*TUB*	0.601	*TUB*	0.194	*TUB*	1.644	*TUB*	2.086	*TUB*	0.426

**Table 4:** Stability analysis of 13 reference genes based on BestKeeper.

Total		Cold-treated		Nacl-treated		PEG-treated		Tissues		Leaves at Different Developmental Stages		Different Borneol Clones	
Gene	SD [±CP]	Gene	SD [±CP]	Gene	SD [±CP]	Gene	SD [±CP]	Gene	SD [±CP]	Gene	SD [±CP]	Gene	SD [±CP]
*ACT7*	0.56	*ACT7*	0.58	*ACT7*	0.52	*ACT7*	0.58	*ACT7*	0.19	*ACT7*	0.79	*ACT7*	0.53
*APT*	0.73	*APT*	0.27	*APT*	1.13	*APT*	0.54	*APT*	0.85	*APT*	0.60	*APT*	0.32
*Cpn60β*	1.00	*Cpn60β*	0.56	*Cpn60β*	0.83	*Cpn60β*	0.38	*Cpn60β*	0.61	*Cpn60β*	1.43	*Cpn60β*	2.37
*EF1α*	0.78	*EF1α*	0.36	*EF1α*	1.28	*EF1α*	0.38	*EF1α*	0.26	*EF1α*	1.03	*EF1α*	0.96
*eIF*-*5A*	0.64	*eIF*-*5A*	0.42	*eIF*-*5A*	0.66	*eIF*-*5A*	0.40	*eIF*-*5A*	0.83	*eIF*-*5A*	0.46	*eIF-5A*	0.63
*GAPDH*	0.96	*GAPDH*	0.37	*GAPDH*	1.21	*GAPDH*	0.57	*GAPDH*	0.32	*GAPDH*	1.80	*GAPDH*	1.45
*Gllα*	0.76	*Gllα*	0.67	*Gllα*	0.75	*Gllα*	0.59	*Gllα*	1.04	*Gllα*	1.09	*Gllα*	0.62
*HIS*	1.09	*HIS*	0.74	*HIS*	1.21	*HIS*	0.71	*HIS*	0.79	*HIS*	1.38	*HIS*	0.10
*RA*	1.87	*RA*	0.53	*RA*	0.68	*RA*	0.80	*RA*	4.74	*RA*	1.20	*RA*	0.89
*RPL27*	0.47	*RPL27*	0.35	*RPL27*	0.44	*RPL27*	0.35	*RPL27*	0.53	*RPL27*	1.01	*RPL27*	0.43
*RPS15*	0.53	*RPS15*	0.55	*RPS15*	0.45	*RPS15*	0.36	*RPS15*	0.56	*RPS15*	0.87	*RPS15*	0.41
*TATA*	0.57	*TATA*	0.48	*TATA*	0.38	*TATA*	0.43	*TATA*	0.52	*TATA*	0.79	*TATA*	0.26
*TUB*	0.83	*TUB*	0.55	*TUB*	0.65	*TUB*	0.37	*TUB*	1.05	*TUB*	2.38	*TUB*	0.80

## Data Availability

Data are contained within the article and Supplementary Materials.

## Supplementary Materials

The following supporting information can be downloaded at: https://www.mdpi.com/article/10.3390/ijms25063500/s1.
