# Supplementary figures and images for: Reference Genes Selection and Validation for Cinnamomum burmanni by Real-Time Quantitative Polymerase Chain Reaction
Source: Int J Mol Sci. 2024 Mar 20;25(6):3500. doi: 10.3390/ijms25063500 (PMC13231827; doi:10.3390/ijms25063500)

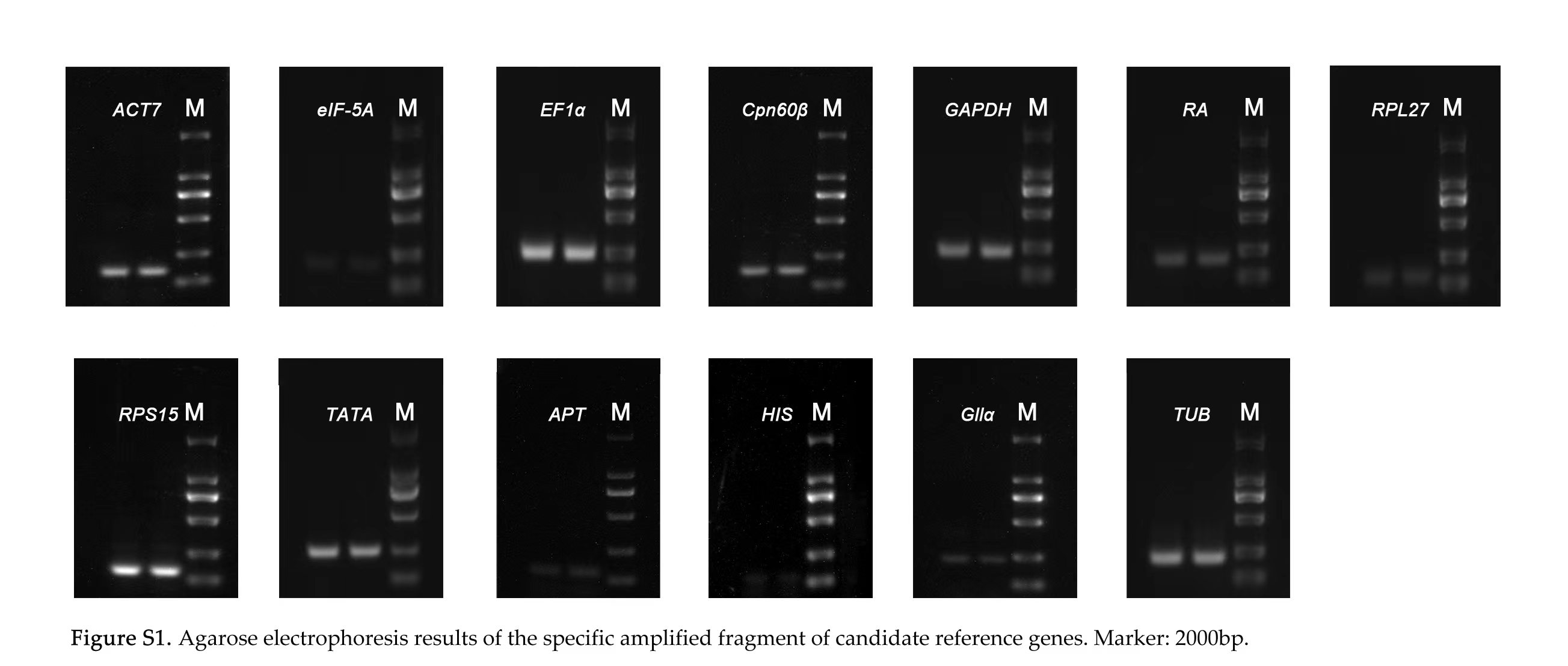

Supplement: Supplementary file 1 [file ijms-25-03500-s001.zip › Figure S1. Agarose electrophoresis results of the specific amplified fragment of candidate reference genes.jpg]

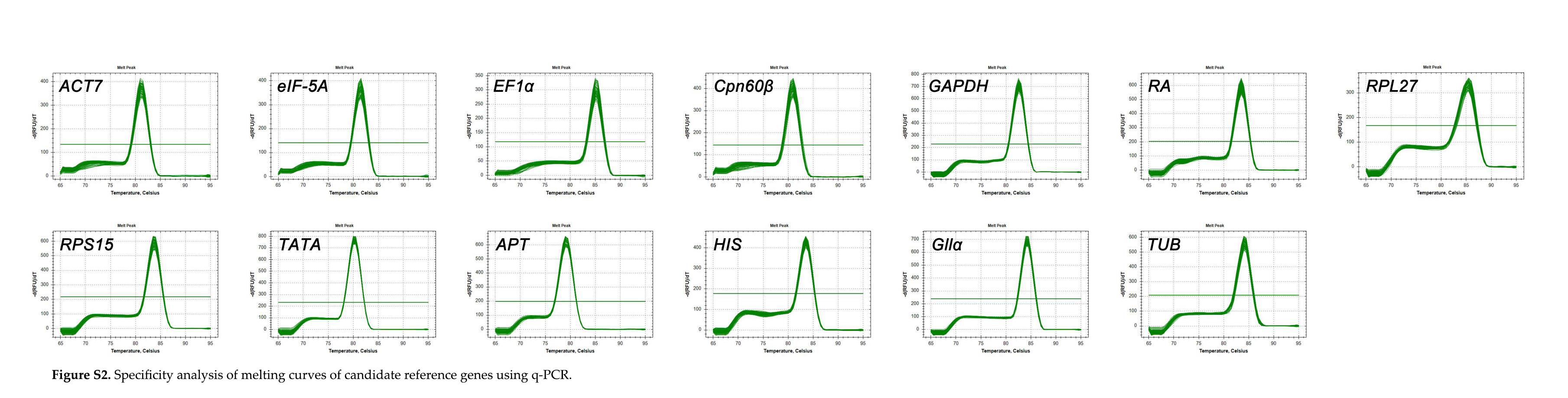

Supplement: Supplementary file 1 [file ijms-25-03500-s001.zip › Figure S2.Specificity analysis of melting curves of candidate reference genes using q-PCR.jpg]

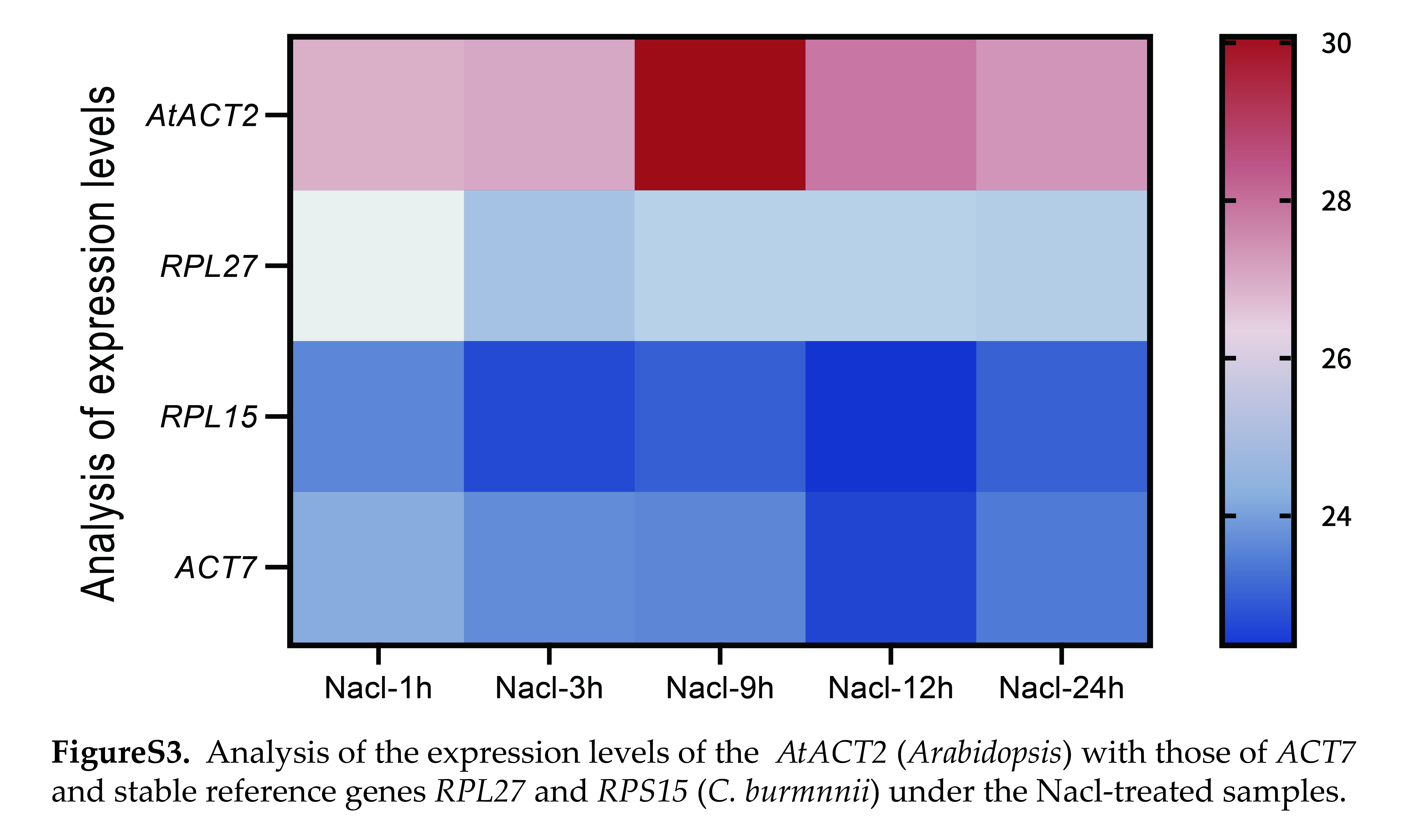

Supplement: Supplementary file 1 [file ijms-25-03500-s001.zip › FigureS3. Analysis of the expression levels of the AtACT2 (Arabidopsis) with those of ACT7 and stable reference genes RPL27 and RPS15 (C. burmnnii) under the Nacl-treated samples.jpg]
